# Dual-Mode Biosensor for Simultaneous and Rapid Detection of Live and Whole *Salmonella typhimurium* Based on Bioluminescence and Fluorescence Detection

**DOI:** 10.3390/bios13030401

**Published:** 2023-03-19

**Authors:** Zhenli Xu, Bailu Liu, Dengfeng Li, Zhenzhong Yu, Ning Gan

**Affiliations:** 1 Faculty of Material Science and Chemical Engineering, Ningbo University, Ningbo 315211, China; 2 Faculty of Marine, Ningbo University, Ningbo 315211, China

**Keywords:** dual-mode biosensor, dead and live bacteria, *Salmonella typhimurium*, magnetic phages, portable ATP bioluminescence, fluorescence meter

## Abstract

Both live and dead *Salmonella typhimurium* (S.T) are harmful to human health, but there are differences in pathological mechanism, dosage, and security. It is crucial to develop a rapid and simultaneous assay to distinguish and quantify live and dead S.T in foods. Herein, one dual-mode biosensor for simultaneous detection of live and dead S.T was fabricated based on two phage probes, using portable bioluminescence and fluorescent meter as detectors, respectively. Firstly, a magnetic phage capture probe (M-P1) and a phage signal tag (P2-S) labeled with SYTO 13 fluorescent dye were prepared, respectively. Both M-P1 and P2-S can specifically conjugate with S.T to form a magnetic sandwich complex. After magnetic separation, the isolated complex can emit a fluorescent signal under an excited 365 nm laser, which can reflect the total amount of S.T. Afterwards, the lysozyme was added to decompose the captured live S.T, which can release ATP and produce a bioluminescent signal corresponding to the live S.T amount. The dead S.T concentration can be deduced by the difference between total and live examples. The detection limit of 55 CFU/mL for total S.T and 9 CFU/mL for live ones was within 20 min. The assay was successfully employed in milk samples and prospectively for on-site screening of other dead and live bacteria, while changing the phages for the targets.

## 1. Introduction

Outbreaks of foodborne pathogens in foods can lead to serious infectious diseases, which have been a great public health threat worldwide [1]. Especially, *Salmonella,* as a gram-negative bacterium, ranks first among all types of bacterial food poisoning [2]. It was estimated that about 93.8 million *Salmonella* infections and 155,000 related deaths occur each year worldwide [3]. Among these, *Salmonella typhimurium* (S.T) is one of the most common serotypes [4]. According to [5], both live and dead S.T are harmful to human health, but there are differences in pathological mechanism, dosage, and security. Live S.T can cause large-scale contamination of foods within a short time, attributed to fast reproduction speed and strong environmental resistance [6,7]. When S.T dies or lyses, it can release endotoxins to cause bacterial virulence and pose a serious health risk to the host [8]. Therefore, effective detection and discrimination of live and dead S.T is essential for selecting an appropriate response protocol in food safety cases. At present, several methods have been developed to detect pathogenic bacteria, such as the plate counting method (the gold standard), enzyme-linked immunosorbent technology (ELISA), polymerase chain reaction (PCR) technology, etc. [9,10,11]. These methods have excellent accuracy, but also some defects. For example, the counting method can only cultivate and calculate the amount of live bacteria, but cannot determine dead ones. Both ELISA and PCR cannot easily discriminate between live and dead S.T. Moreover, their promotion is confined by cumbersome operation, and requirements of expensive equipment and professional operators [12,13].

Since ATP is only found in living bacteria, the ATP bioluminescence method has been widely regarded as a good choice for detection of live bacteria [14,15]. In the presence of oxygen and Mg^2+^, ATP provides energy to luciferin, causing the bioluminescence (BL) reaction catalyzed by luciferase [16]. The bioluminescent signal is directly proportional to the amount of ATP released by the lysing bacteria, allowing high sensitivity and efficient detection of live bacteria [17,18]. Meanwhile, the bioluminescence also exhibits significant advantages of self-illumination without excitation, rapid analysis, high signal noise ratio and sensitivity [19]. In addition, it can also be applied for imaging, enabling visual and accurate observation [20]. Therefore, BL was selected for detecting live S.T in this study. However, ATP exists in all kinds of live bacteria, lacking the recognition ability for bacterial species [21]. ATP based BL assay cannot determine S.T from other kinds of bacteria. In order to enhance the selectivity of BL, fabrication of specific probes is essential to achieve detection of S.T.

Bacteria phages are viruses that attack and eat bacteria. They also have a strong selectivity and binding affinity towards the bacteria strain [22]. Moreover, they are more stable toward the fluctuating of temperature, pH, and ionic strength than antibodies [23]. Therefore, phages are ideal bioreceptors for the construction of highly specific and sensitive probes for pathogen bacteria biosensors. Imai et al [24] synthesized phage-immobilized SiO_2_@AuNP core-shell nanoparticles for the detection of *Staphylococcus aureus* (S.A) and the detection can be completed within 15–20 min with a satisfactory detection limit of 8 × 10^4^ CFU/mL. Chen et al [25] prepared a phage-conjugated magnetic probe for the detection of *Escherichia coli* (*E. coli*) and were able to detect 10^4^ CFU/mL of *E. coli* within 2.5 h with good specificity. Our group [26] has recently fabricated a portable ATP bioluminescence sensor with high specificity for live *Escherichia coli O157:H7* strain, synergistically enhanced by orientated phage-modified stir bar extraction and bio-proliferation. This is suitable for on-site detection of live *E. coli O157:H7* with a low detection limit of 30 CFU/mL within 30 min. In the assay, phage probes were employed in ATP bioluminescence for achieving selective detection of *E. coli O157:H7.* However, these methods could not be used to simultaneously distinguish and detect dead or alive bacteria. In recent years, the fluorescence assay (FL) has been considered an excellent choice for the detection of food-borne pathogenic bacteria, due to its outstanding sensitivity, rapid response, and imaging capacity [27]. Through the fabrication of proper fluorescent probes, bacteria can be detected by FL regardless of whether they are dead or alive [28]. Hence, a dual mode biosensor combining ATP bioluminescence with the fluorescence method was considered as a viable method for the differentiation and detection of dead and live S.T. In order to realize specific detection of S.T, two phage probes with BL and FL signal output were synthesized, respectively. 

**Scheme 1:**
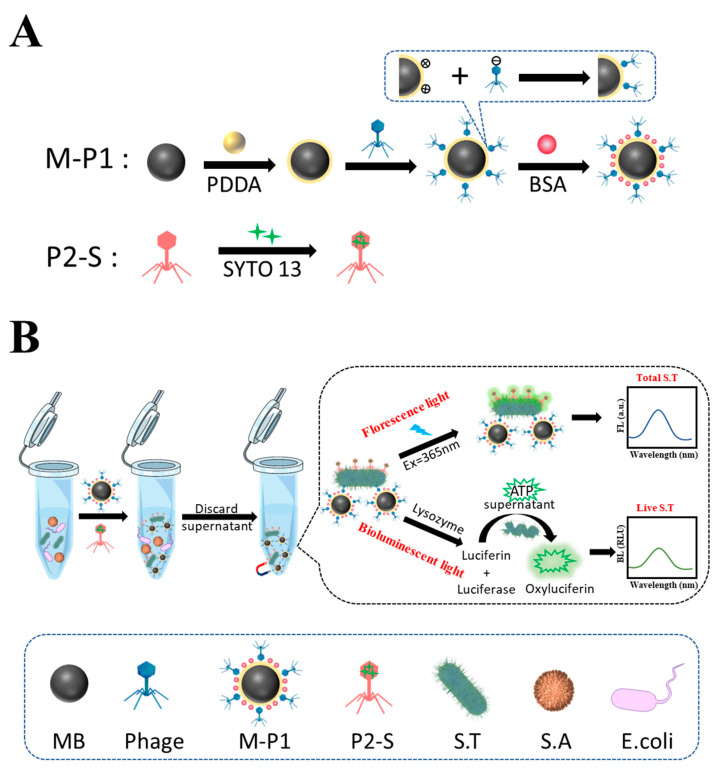
(**A**) The principle of preparation of two phage probes, and (**B**) the dual-mode biosensor for detection of live and whole S.T using the probes.

Most importantly, a one dual-mode biosensor for live and dead S.T was fabricated based on two phage probes using portable bioluminescence and fluorescent meter as detectors, respectively. Firstly, a magnetic phage capture probe (M-P1) and a phage signal tag (P2-S) labeled with SYTO 13 fluorescent dye were prepared, respectively. M-P1 can specifically enrich a trace level of S.T from samples, and P2-S can emit FL signal deriving from the labeled SYTO 13. M-P1 and P2-S can simultaneously and specifically conjugate with S.T, forming a sandwich-like magnetic complex. After magnetic separation, the isolated complex can emit a fluorescent signal under a 365 nm laser, which can reflect the total amount of S.T. Afterwards, the lysozyme was added to disrupt captured live S.T, which can release ATP and produce a bioluminescent signal corresponding to the live S.T amount. The concentration of dead can be deduced by the difference between total and live ones. The probes’ preparation and detection procedures are shown in Scheme 1.

## 2. Materials and Methods

### 2.1. Chemicals and Instrument

Ethylene glycol, anhydrous sodium acetate (NaAc), iron (III) chloride hexahydrate (FeCl_3_∙6H_2_O, 99%), poly dimethyl diallyl ammonium chloride (PDDA, MW: 100,000–200,000 g·mol^−1^, 20 wt% in water), phosphate buffer solution (PBS), ethanol, SYTO 13 fluorescent dye, bovine serum albumin (BSA, MW: 45 KDa), lysozyme, luciferin and luciferase were obtained from Sigma-Aldrich (Shanghai) Trading Co., Ltd. (Shanghai, China). The luria-bertani (LB) broth and agar powder (bacterial grade) were purchased from Hangzhou Microbial Reagent Co., Ltd. (Hangzhou, China). *Vibrio parahemolyticus* (V.P, ATCC 17802), *Salmonella typhimurium* (S.T, ATCC 14028), *E. coli* (CMCC 44484), *Acinetobacter baumannii*, and *Staphylococcus aureus* (S.A, ATCC 25923) were from Shanghai Luwei Technology Co., Ltd. (Shanghai, China). *Salmonella typhimurium* (S.T, ATCC 6538) and its phages were separated and purified by our group and the School of Marine Sciences, Ningbo University.

Fluorescence signal was measured by a fluorescence spectrophotometer from Shimadzu Co., Ltd. (Kyoto, Japan). Bioluminescent signals were gained by a ATP bioluminescence meter purchased from Meicheng Biotechnology Co., Ltd. (Ningbo, China). Fluorescent imaging of probes and magnetic sandwich complexes were characterized by a fluorescence microscope, model MF23-LED, purchased from Mingmei Photoelectric Technology Co., Ltd. (Guangzhou, China). Bioluminescence imaging was displayed by a small animal live imager. Bacteria were incubated by the medical constant temperature incubator purchased from Panasonic Appliances Cold Chain Co., Ltd. (Tokyo, Japan). The surface morphology of probes and magnetic sandwich complexes were analyzed by transmission electron microscope (TEM, JEOL, Tokyo, Japan). Centrifugation was performed using a centrifuge purchased from Sigma Laborzentrifugen GmbH (Berlin, Germany).

### 2.2. The Preparation of M-P1 and P2-S Probes 

The magnetic bead (MB) was synthesized by a hydrothermal method according to the following procedures. Firstly, 3.6 g of anhydrous sodium acetate and 1.3 g of finely ground ferric FeCl_3_**·**6H_2_O were dissolved in 40 mL of ethylene glycol by means of an ultra-sonicator to obtain a yellow viscous solution. The obtained homogeneous solution was sealed in a Teflon-lined stainless-steel autoclave (100 mL volume) and heated at 200 °C for 6 h. After cooling to room temperature, the products were washed three times with ethanol and water, and the final nanoparticles were dried under vacuum for 12 h at 50 °C.

MB-Phage (M-P1) probes were prepared by electrostatic adsorption as follows. 10 mg of MB was dissolved in 1 mL H_2_O and sonicated for 10 min. Then, 1% (wt%) PDDA was added to the Fe_3_O_4_ solution to a final volume of 10 mL, and the reaction was carried out at 2200 rpm for 1 h. At this point, the Fe_3_O_4_ surface was positively charged. After that, the resulting MB@PDDA was repeatedly washed three times by H_2_O and 10^13^ PFU/mL phage was added to it for 1 h at 2200 rpm. The product was separated by a magnet. Due to the negative charge of the phage head, the phage would be fixed directionally on the surface of MB using electrostatic adsorption. The whole process was repeated 4 times with phage addition, each reaction time being 1 h to ensure that as many phages as possible were on the Fe_3_O_4_. Later, the resulting product was repeatedly washed three times by H_2_O, to which 1% (wt%) BSA was added, reacting at 2200 rpm for 1 hour to avoid non-specific adsorption. Finally, the obtained (M-P1) probes were washed 3 times with H_2_O and stored at 4 °C. Phage-SYTO 13 (P2-S) probes were made by mixing 10^13^ phage with 5 mM SYTO 13 dye for 20 min. The mixed product was then passed through a 100 nm filter membrane to remove the free SYTO 13 dye and stored at 4 °C for subsequent use.

### 2.3. Plaque Experiments Using M-P1 and P2-S Probes

Phage plaques are the products obtained from phage engulfment of bacteria, indicating the specificity of the phage for the host bacteria. First, the phage solution obtained from the isolation and purification was diluted in a 10-fold gradient (PBS was used as the dilution medium) to make a series of different concentrations of phage solutions for further use. The prepared LB semi-solid medium (LB mixed with 0.7% agar) was then melted using a far-infrared closed electric oven. Next, 10^8^ CFU/mL S.T (100 μL) and the different concentrations of phage above 100 μL were mixed into 3 mL of the melted LB semi-solid medium. Afterwards, the above mixture was poured onto a Petri dish covered with LB solid medium (LB mixed with 1.5% agar). After the semi-solid medium had solidified, the petri dishes were inverted and placed in an incubator at 37 °C overnight. The appearance of phage plaques in the petri dishes was observed the next day.

### 2.4. Discrimination of Live and Dead S.T by the Dual-Mode Biosensor

1 mL M-P1 probe, 500 μL 10^8^ CFU/mL S.T, and 1 mL P2-S probe were mixed for 10 min at 37 °C. M-P1 and P2-S can be conjugated to S.T, forming a sandwich-like magnetic complex. Later, the sandwich complex was washed 3 times by H_2_O and separated by magnet to remove the free S.T and P2-S probes. Afterwards, the sandwich complex was exposed at 365 nm laser to acquire a fluorescent signal, which can reflect the total amount of S.T. Then, the D-luciferin and luciferase were added to it, at which point the captured live S.T can be disrupted, releasing ATP and thus producing a bioluminescent signal corresponding to the concentration of live S.T, which can be measured by a portable ATP bioluminescence meter. The dead S.T can be calculated by the difference between total and live. Additionally, the ATP of live S.T can be directly and accurately monitored by small animal live images.

### 2.5. Real Samples Pretreatment and Detection

The 200 mL milk samples were purchased from a local supermarket (milk 1 and 2), and 200 mL water samples were collected from tap water (aquatic water 1 and 2). First, samples were filtered by 30–50 μm Whatman filter papers to remove solid particles and other insoluble substances such as the stones and dirt. Then, the supernatant of the sample was added to different concentrations of target S.T. Afterwards, the samples were centrifuged at 5000 r/min for 5 min to precipitate the target bacteria, then the supernatant was discarded. The obtained precipitate was resuspended with 10 mM pH 7.4 PBS (1.0 mL) and further centrifuged. After it was washed 3 times, the precipitate obtained was suspended with 10 mM pH 7.4 PBS, thus to obtain the sample suspension for measurement. Next, the procedure in Section 2.4 was followed to achieve the detection.

## 3. Results and Discussion

### 3.1. Characterization of MB, M-P1 and P2-S Probes

**Figure 1:**
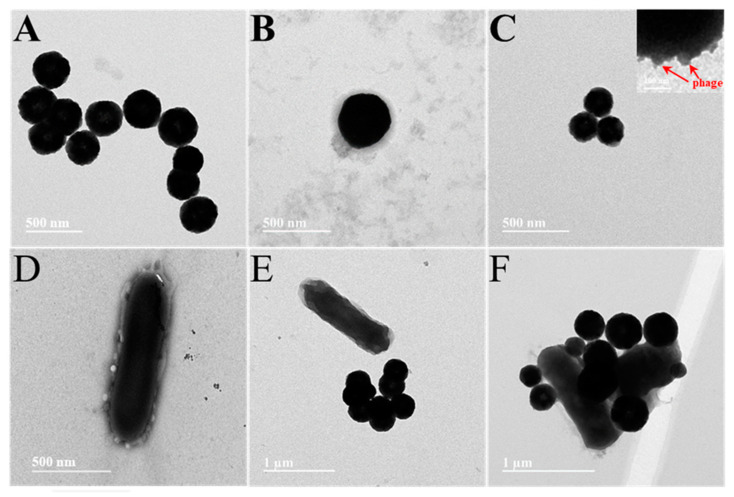
(**A**) pure MBs, (**B**) PDDA-coated MBs, (**C**) M-P1, (**D**) P2-S labelled S.T (**E**) pure MBs captured S.T, (**F**) M-P1 @S.T@P2-S sandwich complex.

To obtain a clear picture of the preparation process of the magnetic complex probes, transmission electron microscopy (TEM) was used to evaluate them. As can be seen in Figure 1A, the MB prepared was of a uniform size of approximately 400 nm. The outer layer of MB in Figure 1B is wrapped by a ring of coating, indicating that PDDA was successfully modified on the MB surface. Many phages were clearly seen in the partial enlargement of Figure 1C attached directionally on top of the MB, demonstrating the successful preparation of M-P1. Figure 1D represents the binding of P2-S to S.T, and many phages can be seen surrounding the S.T. Without the phage, the MB would not bind to the bacteria and could not function as a trap, enrichment, and isolation (Figure 1E). In Figure 1F, a few M-P1 and P2-S can be found tightly adhering to the cell wall of S.T, which suggested a successful preparation of the magnetic complex probe (M-P1@S.T@P2-S), without competing effects between M-P1 and P2-S.

**Figure 2:**
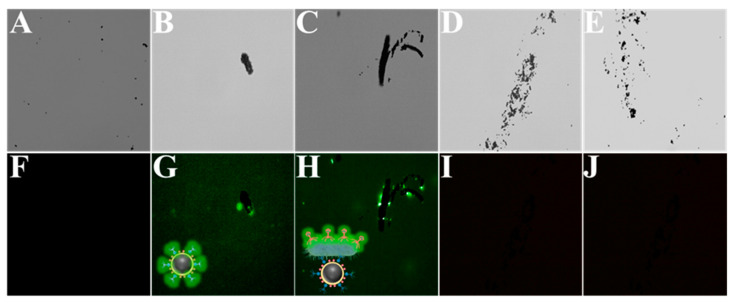
(**A**–**E**) The brightfield of MBs, M-P1, M-P1@S.T@P2-S, M-P1@*E.coli*@P2-S, and M-P1@S.A@P2-S, respectively. (**F**–**J**) correspond to their fluorescent field, respectively.

In order to support the above point of view, a fluorescence microscope was used to further demonstrate the process of preparing magnetic composite probes. The phage was stained with SYTO 13 dye (excitation wavelength at 365 nm and emission wavelength at 520 nm) to obtain images of the successful step-by-step preparation of the magnetic complex (Figure 2). It is clear from the figures that MBs of approximately 400 nm appeared in the field of view (Figure 2A,F) and, once the phage was modified on the MBs, a ring of green fluorescence around the MBs could be seen from the dark-field microscope, declaring the successful preparation of M-P1 (Figure 2B,G). Figure 2C,H distinctly shows that the MBs contain a lot of S.T in the vicinity and that the S.T on the MBs can be seen to produce the fluorescent signal in the dark field, manifesting the successful preparation of the magnetic complex. In addition, the specificity of the prepared probe for S.T was further explored. As can be observed from Figure 2D,I, both the magnetic bead probe and the other bacteria (*E. coli* and S.A) are each free in the field of view and there is no fluorescent signal in the dark field (Figure 2E,J), indicating that the prepared probe has an extremely high specificity for S.T.

### 3.2. The Feasibility of Detection of Live and Dead S.T by the Assay

**Figure 3:**
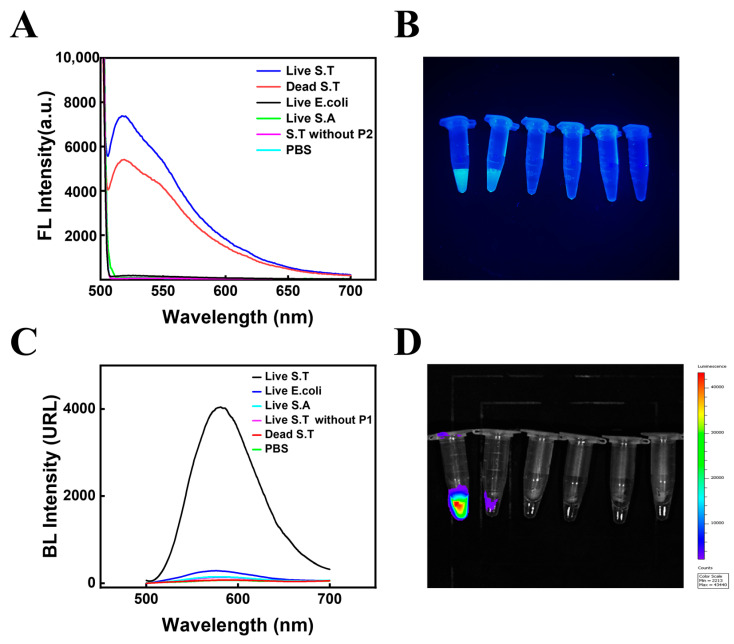
The fluorescence feasibility of the biosensor (**A**) and its physical diagram of fluorescence luminescence (**B**). The bioluminescence feasibility of the biosensor (**C**) and its ATP bioluminescence imaging (**D**). (The concentration of all bacteria was 10^8^ CFU**·**mL^−1^).

Figure 3A shows the fluorescent signals obtained under different conditions, including live and dead 10^8^ CFU**·**mL^−1^ S.T, *E.coli*, S.A, captured and identified by M-P1 and P2-S, as well as live S.T without captured P2, and PBS controls. As can be seen from the figures, for the same concentration of different bacteria, both live and dead S.T produced high fluorescence signal intensities, indicating that the fluorescence method was able to detect both live and dead bacteria and was unable to differentiate between them. In contrast, other miscellaneous bacteria, such as *E. coli* and S.A, had very low and almost absent fluorescence signals, indicating that the phage biosensor has good specificity. Meanwhile, the fluorescence intensities showed that no fluorescence signal was generated without the presence of the P2, further demonstrating the successful modification and specific recognition of the P2-S probe. Figure 3B shows the physical diagram of fluorescence luminescence, the results of which remained consistent with Figure 3A. Moreover, bioluminescence signals were measured under the same conditions (Figure 3C). From the figure, only the live S.T produced high ATP bioluminescence signal intensities when the bacterial concentration was constant, indicating that bioluminescence can only detect live bacteria. For other miscellaneous bacteria, such as *E. coli* and S.A, the ATP bioluminescence signal was extremely low for live bacteria and no bioluminescence signal was present for dead bacteria, again demonstrating the good specificity of this phage biosensor. According to the ATP bioluminescence intensities with and without the presence of P1, it can be seen that when there was no P1 present in the solution, the target bacteria could not be captured and thus the ATP bioluminescence signal generated by bacterial lysis could not be obtained, further demonstrating the successful preparation of this magnetic phage sandwich complex. Figure 3D displays ATP bioluminescence imaging with the same results as Figure 3C.

### 3.3. The Mechanism of the Assay

**Figure 4:**
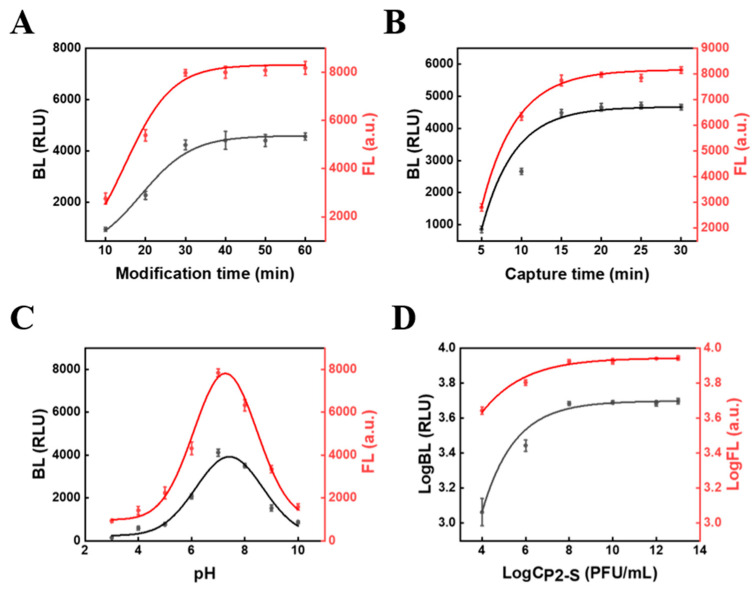
The condition optimization of (**A**) the modification time between P1 and MB@PDDA, (**B**) the time for phage to capture S.T, (**C**) pH, (**D**) and the concentration of P2-S (the concentration of MB was 1 mg/mL). The concentration of S.T was 10^8^ CFU**·**mL^−1^.

The phage can discompose the bacteria after 30 min. Therefore, the effect of phage attachment time on the assay results was considered. In this work, a dual-mode method of FL and BL was used to detect both total and live bacteria and, to ensure the accuracy of the assay, fluorescence was chosen to be measured in the same solution before the bioluminescence signal to avoid errors in the results. Furthermore, as the phage probe can decompose the captured bacteria after 30 minutes, it is easy to have a false positive signal of total S.T concentration within the detection time; thus, the FL signal used to quantify S.T in this work was within 30 min (Figure 4B), avoiding false positive interference.

Because the SYTO dye on the P2-S signal tags will greatly influence the FL signal for detecting live S.T, we also calculated the SYTO amounts on the probes. The following experiment was performed to calculate the amount: 500 µL SYTO 13 with different concentration was mixed with 500 µL 10^9^ PFU/mL phage. The fluorescence light intensity (I) changes were continuously recorded with the change of dye concentration. Because SYTO 13 only emit fluorescence light while combined with phage to form phage@SYTO, the SYTO 13 amount labeled on phage@SYTO can reach saturation after the fluorescence intensity reaches the highest level. According to the amount of the SYTO-13 material at this point, the amount of SYTO 13 in each phage can be calculated. According to the highest peak intensity of SYTO 13 at 0.08 μM, it can be calculated that there were about 48160 SYTO on each P2-S (equation: 8 × 10^−8^M × 5 × 10^−4^L × 6.02 × 10^23^/5 × 10^8^ PFU = 48160 SYTO/phage). The number of M-P1 capture probes around one bacterium can also influence the signal. From the SEM figure, we also found 5–9 M-P1 around one bacterium. This all demonstrated that the assay has dual amplification effects due to many M-P1 surrounding the bacteria and a large number of SYTO on P2-S, all resulting in the high sensitivity of the assay.

### 3.4. Condition Optimization

In order to obtain the best biosensor performance for S.T detection, the modification time between P1 and MB@PDDA was optimized. As depicted in Figure 4A, before 30 min, the signal intensity rapidly increased with time. After 30 min, the signal intensity increased slowly and reached the plateau at 40 min, which indicated that the binding site on MB@PDDA was continuously occupied by P1 as time continued to change, with saturation of MB@PDDA modified by P1 reached at 40 min. Thus, the modification time of 40 min was used for subsequent studies. The time for phage to capture S.T was also optimized (Figure 4B). The signal intensity increased significantly when the capture time was before 15 min, and then levelled off. Subsequent studies have used 15 min as the optimal capture time. The pH was one of the parameters affecting the signal of the biosensor (Figure 4C). With the increase of pH, signal intensity gradually increased and reached the maximum value at pH 7. When the pH was greater than 7, it gradually decreased. This might be because of the reduced growth activity of phages and bacteria in an overly acidic or alkaline environment, resulting in compromised formation of the sandwich complex and thus a reduced signal. The optimum pH of the biosensor was 7 during the whole experiment. Additionally, the concentration of phage probes had an impact on biosensor performance. When the concentration of MB was 1mg/mL, the concentration of P2-S increased from 10^4^ PFU/mL to 10^8^ PFU/mL, and the signal intensity increased rapidly. Signal intensity did not change significantly after a concentration of P2-S more than 10^8^ PFU/mL. Therefore, the optimal concentration of P2-S was 10^8^ PFU/mL.

### 3.5. Detection of the Dual-Mode Biosensor

**Figure 5:**
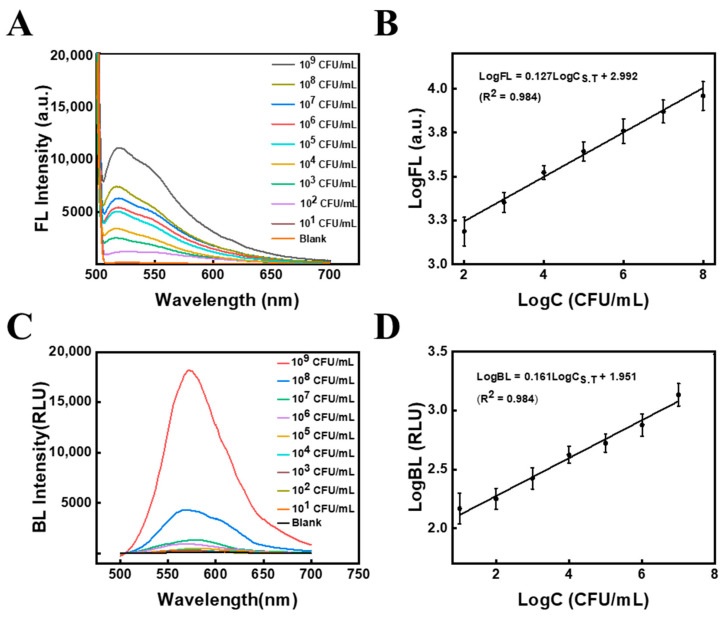
(**A**) Fluorescence intensity signals at different S.T concentrations and (**B**) their corresponding standard curves. (**C**) Bioluminescence intensity signals at different S.T concentrations and (**D**) their corresponding standard curves.

Under the optimal conditions, the biosensor was constructed to detect S.T. In Figure 5A, with different concentrations placed on the biosensor for incubation, FL intensity increased with the increase of concentration. A good linear correlation between the logarithms of FL intensity and S.T concentration was obtained (Figure 5B) with the linear ranging from 10^2^ to 10^8^ CFU/mL, and the linear regression equation of the sensor was LogFL = 0.127 Log C_S.T_ + 2.992. The correlation coefficient (R^2^) was 0.984. In the meantime, as shown in Figure 5C, BL intensity also increased with the increase of S.T concentration. When the linear ranging from 10^1^ to 10^7^ CFU/mL, the linear regression equation was LogBL = 0.161 LogC_S.T_ + 1.951, and R^2^ was 0.984 (Figure 5D). The calculated result showed that the detection limit (LOD) was 55 CFU/mL and 9 CFU/mL, demonstrating that the biosensor possessed a low detection limit and a wide linear range.

### 3.6. The Fabrication of the Portable FL Detector

**Figure 6:**
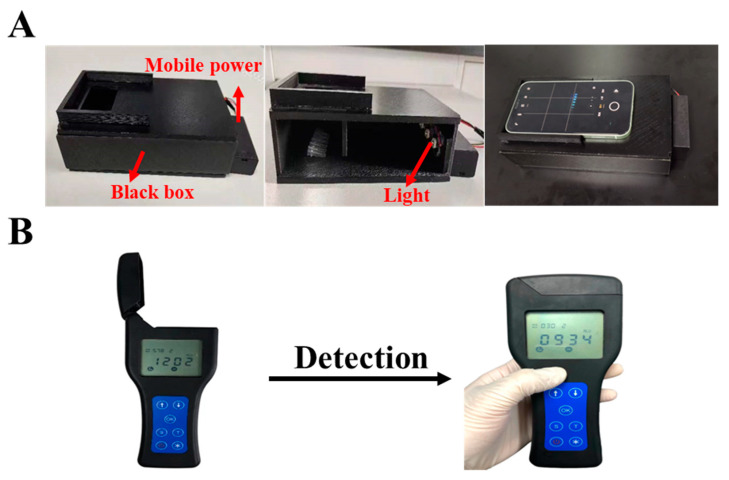
(**A**) The configuration of self-made FL detector. (**B**) The portable ATP bioluminescent meter.

The fluorescent assay was visual and rapid. A portable FL detector was fabricated to perform the detection, as shown in Figure 6A. Its configuration was described as following: the FL detector consists mainly of a black box and a light source. The overall size of the black box is 15  ×  8  ×  5 cm (Length × width × height), and the observation window is 6 × 4 cm (Length × width). The detection window was at the top and covered by a mobile phone for convenient observation to eliminate other distractions. A fixing bracket with 7 × 0.5 × 1 cm (Length × width × height) was designed near the detection window to ensure that the mobile phone was in the same position each time the picture was taken, reducing errors. Meanwhile, the plate on the left side of the black box had been designed as a pull-out, which aimed to facilitate the insertion and removal of samples. The light’s power was 3 W. The whole design can make sure that all samples can be fully exposed to the light source without distance difference and avoid the interference of background light to offer effective visual detection results. The commercial portable BL detector was employed to analyze live S.T (Figure 6B). By mixing the sandwich complex with a luminescent solution containing luciferin, luciferase, and lysozyme for 5 min and then inserting the reaction solution into the portable BL detector, the bioluminescence intensity can be measured within 15 s. Both FL and BL methods are simple to operate, have short testing times and are suitable for on-site testing.

### 3.7. Specificity of the Biosensor

**Figure 7:**
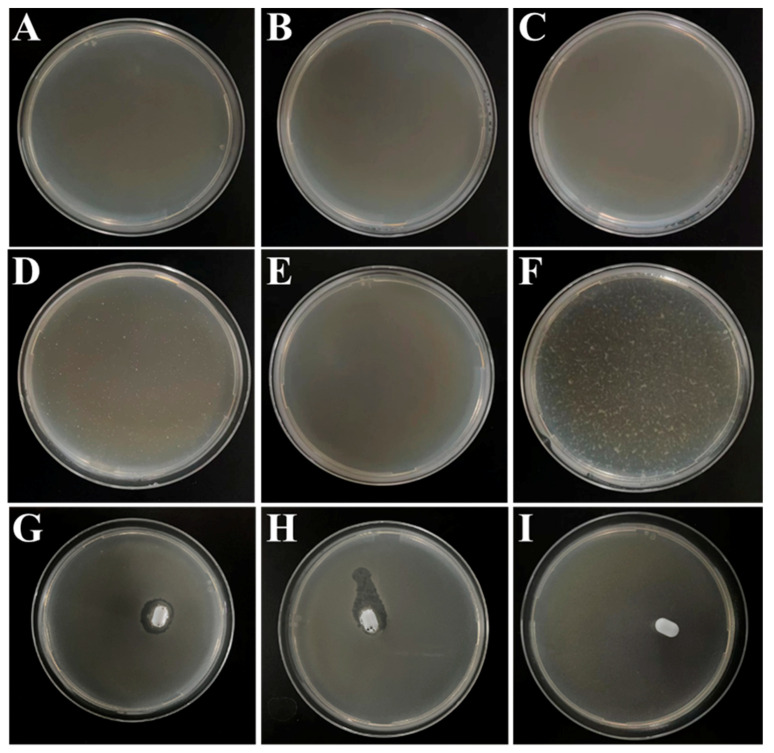
Plaque produced after dilution of phage stock solution by 10^6^ times for (**A**) *E. coli*, (**B**) Acinetobacter Baumann, (**C**) V.P, (**D**) S.A, (**E**) L.M, and (**F**) S.T with P1, (**G**) P1, (**H**) P2, and (**I**) PBS with MB.

Since multiple bacterial pathogens and target bacteria coexist in most instances, the specificity of the sensor should be confirmed. In this work, the specificity is mainly derived from the phage, and can therefore be demonstrated by phage plaque experiments. As can be seen in Figure 7, many plaques appeared only in the plates containing S.T (Figure 7F), while no phage plaques appeared in the plates containing *E.coli*, Acinetobacter Baumann, V.P, S.A and L.M (Figure 7A–E), which suggested that the phages screened were highly specific for S.T. The effect of magnetic composite probes on phage specificity was also explored (Figure 7G–F). As seen in the figures, phage plaques were only produced in the presence of phage (Figure 7G,H) and only around the magneton, and no phage plaques were produced without phage (Figure 7I), demonstrating that the MB would not affect the specificity of the phage and providing further evidence that the phage was successfully modified on the MB.

### 3.8. The Comparison with Other Assays

For sake of explaining the advantages of this work, some previous methods of S.T detection were listed for comparison (Table 1). In comparison to the works of Chen [29], Bu [30] and Wei et al [31], although their work had an extremely low detection limit, the detection time was quite long and not suitable for in situ detection. The work of Zhuang’s group took only 3 min to detect, but the detection limit was as high as 10^3^ CFU/mL and with a lower sensitivity [32]. On top of that, this work can detect both live and dead S,T within 20 min, using methods of fluorescence and bioluminescence, with short detection time, low detection limit and high sensitivity.

### 3.9. Real Sample Detection

In order to further determine the applicability of the method in actual samples, the different milk and water samples were used as actual samples to be analyzed for its detection conditions. The recoveries of the four samples were tested by standard addition method and the results were summarized in Table 2 and Table 3. The recovery rates of S.T in both milk and both water samples were all above 92%, and the RSD values were all less than 8%. The above results indicated that the biosensor was comparably reliable for determination both dead and live S.T residues in actual samples.

**Figure 8:**
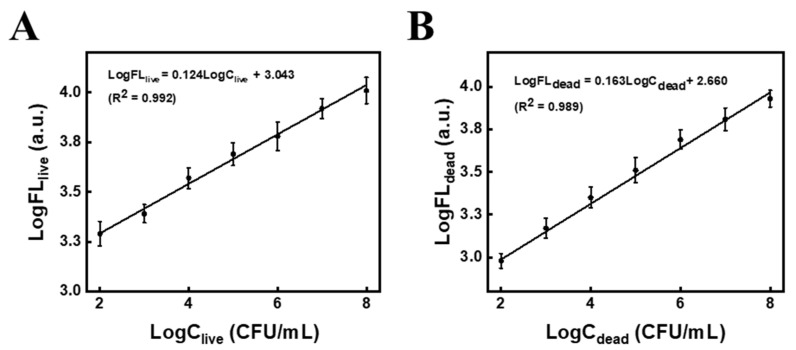
Fluorescence calibration curves at different concentrations of (**A**) live S.T and (**B**) dead S.T.

It is worth noting that the difference was used to discriminate between live and total S.T to calculate dead S.T, while a sample with a different ratio between live and dead cells should not be correctly quantified if the difference is calculated, so it should require a differential calibration of live and dead S.T. The detailed steps are as follows: first, we have remeasured and plotted the FL calibration curves for live and dead S.T (Figure 8), and BL calibration curves for live S.T. The relevant regression equation are as follows:

Log FL_live_ = 0.124 Log C_live_ + 3.043, R^2^ = 0.992 (1)

Log FL_dead_ = 0.163 Log C_dead_ + 2.660, R^2^ = 0.989 (2)

Log BL_live_ = 0.161 Log C_live_ + 1.951, R^2^ = 0.984 (3)

where FL_live_ is the calibration FL for live S.T, FL_dead_ is the calibration FL for dead S.T, and BL_live_ is the calibration BL for live S.T. 

Then, the spiked sample with mixture of living and dead S.T. was measured by FL_total_ and BL_live_, respectively. From Equation (3), the concentration of living S.T (C_live_) was obtained. Next, the Clive was plug into Equation (1), the FL_live_ of live ones can be calculated. According to the equation: FL_totoal_ = FL_live_ + FL_dead_ (4), the FL_dead_ can be acquired. After that, the FL_dead_ was fitted into Equation (2), and the concentration of dead S.T (C_dead_) was obtained. Based on the above calculation, the concentration of live and dead S.T in real samples was calculated (Table 2). 

## 4. Conclusions

A one dual-mode biosensor for live and dead S.T was fabricated based on a couple of phage probes using portable bioluminescence and fluorescent meter as detectors, respectively. To fulfil the purpose, a magnetic phage capture probe (M-P1) and a phage signal tag (P2-S) labeled with SYTO 13 fluorescent dye were prepared, respectively. The total can be reflected by the FL signal from P2-S, while live bacteria can be quantified based on ATP bioluminescence. The dead S.T concentration can be deduced by the difference between total and live. Both methods can be used for quantification by the portable meter, with a detection limit of 55 CFU/mL for total S.T and 9 CFU/mL for live within 20 min. The assay was successfully employed in milk samples and prospectively can be utilized for on-site screening other dead and live bacteria, while changing the phages for the target.

## Figures and Tables

**Table 1:** Comparison of methods to detect S.T.

Methods	LOD ^a^ (CFU·mL^−1^)	Linear Range (CFU·mL^−1^)	Time (min)	Identifications of Live/Dead Bacteria	Ref.
multimode dot-filtration immunoassay	10	10^1^~10^6^	20	No	[33]
UCNPs PCR fluorescence sensor	11	11~1.14 × 10^9^	90	No	[29]
Electrochemical immunoassay	3	10~10^7^	90	No	[30]
Fluorescence biosensor	10^3^	7.0 × 10^3^~3.0 × 10^8^	3	No	[32]
Colorimetric	7	10~10^7^	45	No	[31]
Fluorescence and Bioluminescence	50	10^1^~10^8^	20	Yes	This work

^a^ Limit of detection.

**Table 2:** Detection of total S.T in real samples by FL (*n* = 3).

Method	Sample	Added (CFU·mL^−1^)	Measured (CFU·mL^−1^)	C_live_ (CFU·mL^−1^)	C_dead_ (CFU·mL^−1^)	Recovery (%)	RSD (%)
FL	Milk 1	0	-			-	-
		10^3^	(0.927 ± 0.055) × 10^3^	1.202 × 10^−5^	9.120 × 10^−14^	92.8	5.5
		10^6^	(0.946 ± 0.032) × 10^6^	0.096	2.799 × 10^2^	94.6	3.4
	Milk 2	0	59 ± 10	1.585 × 10^−9^	3.715 × 10^−18^	-	5.4
		10^3^	(1.045 ± 0.068) × 10^3^	1.202 × 10^−5^	1.888 × 10^−13^	104.5	6.8
		10^6^	(1.096 ± 0.025) × 10^6^	0.096	6.918 × 10^2^	109.6	2.5
	Water 1	0	-			-	-
		10^3^	(0.932 ± 0.047) × 10^3^	1.202 × 10^−5^	9.333 × 10^−14^	93.2	4.7
		10^6^	(0.958 ± 0.061) × 10^6^	0.096	3.013 × 10^2^	95.8	6.1
	Water 2	0	-			-	-
		10^3^	(0.951 ± 0.048) × 10^3^	1.202 × 10^−5^	1.059 × 10^−13^	95.1	4.8
		10^6^	(0.979 ± 0.064) × 10^6^	0.096	3.467 × 10^2^	98.0	6.4

**Table 3:** Detection of live S.T in real samples by BL (*n* = 3).

Method	Sample	Added (CFU·mL^−1^)	Measured (CFU·mL^−1^)	Recovery (%)	RSD (%)
BL	Milk 1	0	-	-	-
		10^3^	(0.966 ± 0.035) × 10^3^	96.5	3.5
		10^6^	(0.948 ± 0.071) × 10^6^	94.8	7.1
	Milk 2	0	43 ± 6	-	4.9
		10^3^	(0.974 ± 0.023) × 10^3^	97.4	2.3
		10^6^	(0.983 ± 0.076) × 10^6^	98.3	7.6
	Water 1	0	-	-	-
		10^3^	(0.992 ± 0.077) × 10^3^	99.2	7.7
		10^6^	(1.014 ± 0.052) × 10^6^	101.4	5.2
	Water 2	0	-	-	-
		10^3^	(1.027 ± 0.059) × 10^3^	102.7	5.9
		10^6^	(0.988 ± 0.038) × 10^6^	98.8	3.8

## Data Availability

All research data is included in this article.

## References

[1] Yang T., Luo Z., Bewal T., Li L., Xu Y., Jafari S.M., Lin X. (2022). When smartphone enters food safety: A review in on-site analysis for foodborne pathogens using smartphone-assisted biosensors. Food Chem..

[2] Li Q., Zhang M., Zhang Q., Zhu Z., Guo Z., Li J., Xu W., Zhu J., Yao Y., Li Z. (2023). An autonomous synthetic DNA machine for ultrasensitive detection of Salmonella typhimurium based on bidirectional primers exchange reaction cascades. Talanta.

[3] Majowicz S.E., Musto J., Scallan E., Angulo F.J., Kirk M., O’Brien S.J., Jones T.F., Fazil A., Hoekstra R.M., Studies International Collaboration on Enteric Disease ’Burden of Illness (2010). The Global Burden of Nontyphoidal Salmonella Gastroenteritis. Clin. Infect. Dis..

[4] De Oliveira T.R., Martucci D.H., Faria R.C. (2018). Simple disposable microfluidic device for Salmonella typhimurium detection by magneto-immunoassay. Sens. Actuators B Chem..

[5] Zhang P., Liu H., Li X., Ma S., Men S., Wei H., Cui J., Wang H. (2017). A label-free fluorescent direct detection of live Salmonella typhimurium using cascade triple trigger sequences-regenerated strand displacement amplification and hairpin template-generated-scaffolded silver nanoclusters. Biosens. Bioelectron..

[6] Chapman B., Gunter C. (2018). Local Food Systems Food Safety Concerns. Microbiol. Spectr..

[7] Du H., Zhang X., Yao M., Yang Q., Wu W. (2022). Aptamer-guided luminous microsphere for anchoring and lightening Salmonella enterica serovar Typhimurium. Sens. Actuators B Chem..

[8] Dufour N., Delattre R., Ricard J.D., Debarbieux L. (2017). The Lysis of Pathogenic Escherichia Coli by Bacteriophages Releases Less Endotoxin Than by Beta-Lactams. Clin. Infect. Dis..

[9] Yin W., Zhu L., Xu H., Tang Q., Ma Y., Chou S.-H., He J. (2022). Bio-hybrid nanoarchitectonics of nanoflower-based ELISA method for the detection of Staphylococcus aureus. Sens. Actuators B Chem..

[10] Kumaragurubaran N., Arul P., Huang S.-T., Huang C.-H., Fang S.-B., Lin Y.-H. (2023). Nanocatalyst coupled with a latent-ratiometric electrochemical switch for label-free zero-tolerance rapid detection of live Salmonella in whole blood samples. Sens. Actuators B Chem..

[11] Chen F.-E., Trick A.Y., Hasnain A.C., Hsieh K., Chen L., Shin D.J., Wang T.-H. (2022). Ratiometric PCR in a Portable Sample-to-Result Device for Broad-Based Pathogen Identification. Anal. Chem..

[12] Du Z., Wang Y., He D., Xu E., Chai Q., Jin Z., Wu Z., Cui B. (2022). Iproving the sensitivity of lateral flow immunoassay for Salmonella typhimurium detection via flow-rate regulation. Food Chem..

[13] Ji L., Zhang L., Yang H., Liang S., Pan J., Zou Y., Li S., Li Q., Zhao S. (2022). Versatile Au@Ru nanocomposites for the rapid detection of Salmonella typhimurium and photothermal sterilization. J. Colloid Interface Sci..

[14] Kim S.U., Jo E.-J., Noh Y., Mun H., Ahn Y.-D., Kim M.-G. (2018). Adenosine Triphosphate Bioluminescence-Based Bacteria Detection Using Targeted Photothermal Lysis by Gold Nanorods. Anal. Chem..

[15] Finger S., Wiegand C., Buschmann H.-J., Hipler U.-C. (2013). Antibacterial properties of cyclodextrin–antiseptics-complexes determined by microplate laser nephelometry and ATP bioluminescence assay. Int. J. Pharm..

[16] Ding B.-W., Liu Y.-J. (2017). Bioluminescence of Firefly Squid via Mechanism of Single Electron-Transfer Oxygenation and Charge-Transfer-Induced Luminescence. J. Am. Chem. Soc..

[17] Chen F., Warnock R.L., Van Der Meer J.R., Wegner S.V. (2020). Bioluminescence-Triggered Photoswitchable Bacterial Adhesions Enable Higher Sensitivity and Dual-Readout Bacterial Biosensors for Mercury. ACS Sens..

[18] He Y., Wang M., Fan E., Ouyang H., Yue H., Su X., Liao G., Wang L., Lu S., Fu Z. (2017). Highly Specific Bacteriophage-Affinity Strategy for Rapid Separation and Sensitive Detection of Viable *Pseudomonas aeruginosa*. Anal. Chem..

[19] Xu Q., Ma F., Huang S.-Q., Tang B., Zhang C.-Y. (2017). Nucleic Acid Amplification-Free Bioluminescent Detection of MicroRNAs with High Sensitivity and Accuracy Based on Controlled Target Degradation. Anal. Chem..

[20] Gregor C., Pape J.K., Gwosch K.C., Gilat T., Sahl S.J., Hell S.W. (2019). Autonomous bioluminescence imaging of single mammalian cells with the bacterial bioluminescence system. Proc. Natl. Acad. Sci. USA.

[21] Xu Z., Zeng G., Liu Y., Zhang X., Cheng J., Zhang J., Ma Z., Miao M., Zhang D., Wei Y. (2018). Monitoring mitochondrial ATP in live cells: An ATP multisite-binding fluorescence turn-on probe. Dye. Pigment..

[22] Ertürk G., Lood R. (2018). Bacteriophages as biorecognition elements in capacitive biosensors: Phage and host bacteria detection. Sens. Actuators B Chem..

[23] Richter Ł., Janczuk-Richter M., Niedziółka-Jönsson J., Paczesny J., Hołyst R. (2018). Recent advances in bacteriophage-based methods for bacteria detection. Drug Discov. Today.

[24] Imai M., Mine K., Tomonari H., Uchiyama J., Matsuzaki S., Niko Y., Hadano S., Watanabe S. (2019). Dark-Field Microscopic Detection of Bacteria using Bacteriophage-Immobilized SiO_2_@AuNP Core–Shell Nanoparticles. Anal. Chem..

[25] Chen J., Alcaine S.D., Jiang Z., Rotello V.M., Nugen S.R. (2015). Detection of *Escherichia coli* in Drinking Water Using T7 Bacteriophage-Conjugated Magnetic Probe. Anal. Chem..

[26] Cao C., Wang M., Zhang D., Yu S., Xie H., Wang Q., Yu Z., Gan N. (2023). Portable ATP bioluminescence sensor with high specificity for live Escherichia coli O157:H7 strain synergistically enhanced by orientated phage-modified stir bar extraction and bio-proliferation. Biosens. Bioelectron..

[27] Fu L., Qian Y., Zhou J., Zheng L., Wang Y. (2020). Fluorescence-based quantitative platform for ultrasensitive food allergen detection: From immunoassays to DNA sensors. Compr. Rev. Food Sci. Food Saf..

[28] Zhu K., Qin T., Zhao C., Luo Z., Huang Y., Liu B., Wang L. (2018). A novel fluorescent turn-on probe for highly selective detection of nitroreductase in tumor cells. Sens. Actuators B Chem..

[29] Chen M., Han L., Zhou D., Kong L., Pan L., Tu K. (2022). Amplified UCNPs-Mitoxantrone dihydrochloride fluorescence PCR sensor based on inner filter for ultrasensitive and rapid determination of Salmonella typhimurium. Sens. Actuators B Chem..

[30] Bu S.-J., Wang K.-Y., Liu X., Ma L., Wei H.-G., Zhang W.-G., Liu W.-S., Wan J.-Y. (2020). Ferrocene-functionalized nanocomposites as signal amplification probes for electrochemical immunoassay of Salmonella typhimurium. Microchim. Acta.

[31] Wei S., Su Z., Bu X., Shi X., Pang B., Zhang L., Li J., Zhao C. (2022). On-site colorimetric detection of Salmonella typhimurium. NPJ Sci. Food.

[32] Zhuang Q.Q., He S.B., Jiang Y.C., Huang K.Y., Xu Y.Y., Peng H.P., Deng H.H., Chen W. (2022). Immunofluorescent-Aggregation Assay Based on Anti-Salmonella Typhimurium Igg-Auncs, for Rapid Detection of Salmonella Typhimurium. Mikrochim. Acta.

[33] Gao L., Xu X., Liu W., Xie J., Zhang H., Du S. (2022). A sensitive multimode dot-filtration strip for the detection of Salmonella typhimurium using MoS_2_@Fe_3_O_4_. Microchim. Acta.

